# Recent Progress on Gold-Nanocluster-Based Fluorescent Probe for Environmental Analysis and Biological Sensing

**DOI:** 10.1155/2019/1095148

**Published:** 2019-01-02

**Authors:** Mingxian Liu, Fenglin Tang, Zhengli Yang, Jing Xu, Xiupei Yang

**Affiliations:** College of Chemistry and Chemical Engineering, Chemical Synthesis and Pollution Control Key Laboratory of Sichuan Province, China West Normal University, Nanchong 637000, China

## Abstract

Gold nanoclusters (AuNCs) are one of metal nanoclusters, which play a pivotal role in the recent advances in the research of fluorescent probes for their fluorescence effect. They are favored by most researchers due to their strong stability in fluorescence and adjustability in fluorescence wavelength when compared to traditional organic fluorescent dyes. In this review, we introduce various synthesis strategies of gold-nanocluster-based fluorescent probes and summarize their application for environmental analysis and biological sensing. The use of gold-nanocluster-based fluorescent probes for the analysis of heavy metals and inorganic and organic pollutants is covered in the environmental analysis while biological labeling, imaging, and detection are presented in biological sensing.

## 1. Introduction

Nanomaterials possess quite different properties such as quantum size effect [1], easy synthesis, and biocompatibility [2]. Metal nanoclusters are one of them, which have been successfully produced, including silver clusters [3], platinum clusters [4], palladium clusters [5], copper clusters [6], and core-shell bimetallic nanoclusters (platinum-copper [7], copper-ruthenium [8], and palladium-copper [9]). In recent years, metal nanoclusters have been used in catalysis [10], optics [11], electrochemistry [12], biotechnology [13], and other fields. The nanogold particles, one of the metal nanoclusters, are referred to as colloidal gold because the water sol is generally dispersed and the size ranges between 1 and 100 nm.

Fluorescent gold nanoclusters (AuNCs) or nanodots (NDs) with sizes less than 3 nm are a specific type of nanogold particles. In recent years, a variety of gold nanoclusters have been prepared and their potential application has been demonstrated in areas such as analytical chemistry, biology, medicine, and environmental science. For more than ten years, researchers have characterized metallic nanoclusters in terms of size, morphology, and surface modification groups and have made continuous improvements and development of their synthesis [14–16]. The synthesis of gold nanoparticles is mainly divided into physical and chemical methods. Physical preparation methods of gold particles are mainly performed by employing a variety of dispersion technologies to promote gold directly into gold nanoparticles, including ultrasonication [17], laser ablation [18], and other methods. With physical methods, the control of the size of nanoparticles is full of challenges. In contrast, the size of the gold nanoparticles can be controlled and achieved by applying the designated reaction conditions in chemical methods in which the gold compounds are used as raw materials via a reduction reaction. Chemical methods include the aqueous phase oxidation-reduction method [19], seeding method [20], microemulsion method [21], as well as the template method [22]. Compared to the physical method, the chemical one has gained growing popularity because of its simple operation, lower cost, ease of control of the size of the synthesized nanoparticles, and the possibility of surface modification.

The most classic method of synthesis is the citric acid method, which enables the synthesis to be conducted in a water phase, making it widely applicable. With this method, HAuCl_4_ is reduced by citric acid (CA) in the water phase at a high temperature. The two adjacent carboxyl groups of CA impact the surface of gold nanoparticles (AuNPs) by a coordination effect, and the excess carboxyl groups are spread outward to promote stable dispersion of AuNPs [23]. The thiol method, also known as the Brust–Schiffrin process, generally occurs in a two-phase system composed of water and toluene to facilitate the synthesis of monodisperse AuNPs. In 1994, Brust et al. were the first to synthesize monodisperse gold nanoclusters protected by a mercapto ligand in a two-phase liquid–liquid system [24]. Later on, Negishi et al. improved this method [25].

In addition, the nanoparticles can be stabilized by encapsulation within the dendrimers effectively because of their uniform composition and structure [26, 27], which have become a template for the effective synthesis of AuNPs.

In recent years, the development of nanomaterials, especially nanoclusters [28, 29], has enriched the research of nanomaterials science. The preparation of related functional materials [30, 31] using gold nanoparticles has received much attention for their ease of preparation and modification, high density, and dielectric constant. Gold nanoclusters are widely used in many fields such as fluorescence detection [32–34], biomarkers [35, 36], and catalysis [37, 38] because of their strong fluorescence stability, good biocompatibility, low cytotoxicity, and large Stoke's shift. However, the mechanics of gold particles and structures are still full of challenges because there are still some unreachable obstacles for researchers to explore their nature and formation. For example, most of the synthesized gold nanoparticles are polydisperse, and it may be necessary to obtain monodisperse ones for the establishment of the relationship between their function and structures. There are some reports on the methods to achieve monodisperse gold nanoclusters by tuning the synthesis conditions. Our group had separated water-soluble gold nanoclusters (AuNCs) by sequential size-selective precipitation (SSSP) and characterized the fractions with mass spectrometry (MS). The AuNCs could be precipitated and separated successively from larger to smaller ones, and Au_38_(SR)_18_, Au_28_(SR)_15_, Au_18_(SR)_12_, and Au_11_(SR)_8_ were obtained [39].

### 1.1. Application of Gold Nanoclusters in Fluorescent Probes for Environmental Analysis

With the development of industry, possible pollutants may be introduced into the environment and accumulated to a certain extent detrimental to both the environment and human beings [40]. The implementation of real-time monitoring measures is a prerequisite for environmental protection and disease prevention [41, 42]. Heavy metal ions such as Hg^2+^ [43], Cd^2+^ [44], Pb^2+^ [45], and Cu^2+^ [46] may easily bind with proteins, enzymes, and nucleic acids, which may cause changes in the biological functions of these substances [47]. Therefore, it is necessary to have a method to rapidly detect these contaminants with sufficient sensitivity. In recent years, the appearance and application of nanomaterials and fluorescent probes have provided new ideas for this approach. AuNCs have attracted the attention of many researchers and have been used in fluorescence sensors for the detection of environmental pollutants [48, 49].

**Figure 1:**
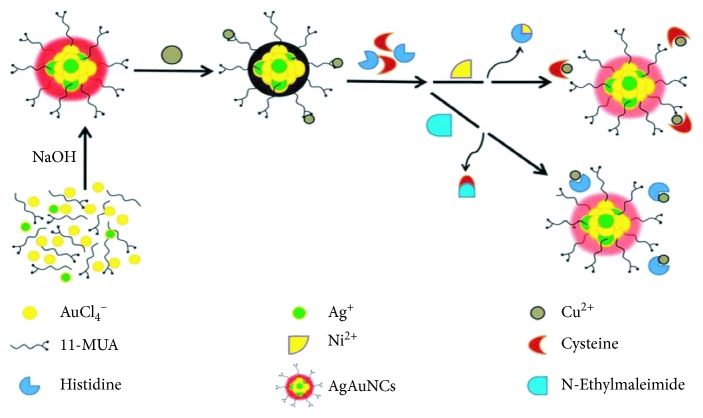
The schematic representation of the synthesis strategy for AgAuNCs@11-MUA preparation and amino acid-sensing mechanism. (Copyright @2017 American Chemical Society).

In 2014, Anitha Senthamizhan and Tamer Uyar synthetized AuNC fluorescent probes protected by bovine serum albumin (BSA) and achieved the visual detection of Hg^2+^ successfully. When the composite nanofibers encountered Hg^2+^, the visible fluorescence signal would change significantly. Therefore, the sensitive detection of Hg^2+^ could be realized by observing the color change of the composite. Meanwhile, the established method can be applied on other common toxic metallic interferences (Pb^2+^, Mn^2+^, Cu^2+^, Ni^2+^, Zn^2+^, and Cd^2+^) in the water of environmental samples and with a good specificity [50]. Besides this, there are many ways to detect Hg^2+^ by fluorescent probes; Guanghua Zhao prepared *β*-Lg-AuNCs using *β*-lactoglobulin (Lg) as a biological template. The *β*-Lg-AuNCs were reported with high intensity of fluorescence and water solubility and high sensitivity and selectivity for the detection of Hg^2+^ in aqueous media. In another study, *β*-lactoglobulin-stabilized AuNCs were synthesized and used as fluorescence sensors to determine Hg^2+^ with high sensitivity in urine, beverages, and serum samples [51]. Zhijun Chen et al. synthesized highly fluorescent gold nanoclusters by using a proteolytic enzyme, a-chymotrypsin A (CTRA), as both the stabilizing and reducing agents and applied them for mercury ion detection with both the fluorometric method (UV light) and the colorimetric one (the naked eye). For the possible specific detection mechanism of mercury ions, they explained that mercury ions can specifically quench the red fluorescent AuNCs@CTRA band and selectively stop green-band formation on the membrane through inhibition of the peroxidase mimic activity of AuNCs@CTRA toward the 2,2'-azino-bis(3-ethylbenzothiazoline-6-sulphonic acid) (ABTS) substrate in a concentration-dependent manner [52]. Similarly, preliminary progress has also been made in the detection of Cu^2+^ by fluorescent probes [53, 54]. Xiurong Yang et. al. employed 11-mercaptoundecanoic acid (11-MUA) as a reducing agent and protecting ligand to prepare fluorescent Ag/Au bimetallic nanoclusters (namely AgAuNCs@11-MUA) at room temperature (Figure 1). Interestingly, they found that the fluorescence of AgAuNCs@11-MUA can be selectively quenched by Cu^2+^ ions, and the nonfluorescence off-state can be effectively switched on by adding histidine and cysteine. This phenomenon is also exploited further as an integrated logic gate, and a specific fluorescence activation assay has been designed and established to selectively and sensitively detect histidine and cysteine [55].

**Figure 2:**
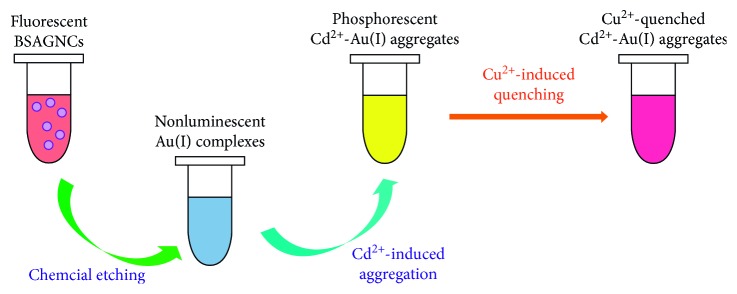
The scheme of aggregation-induced emission and fluorescence quenching mechanism.

**Figure 3:**
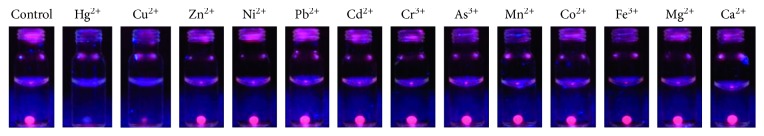
Photographs of vials containing hydrogel beads placed in 1 ppm concentration of various heavy-metal ions. (Copyright @2017 American Chemical Society).

The method of synthesis of AuNCs plays an important role in their application for their different structures and properties. Apart from the above direct synthesis, chemical etching is also a good way to get AuNCs. Chemical etching of gold by thiols has been known to be capable of generating nonluminescent gold (I) complexes and these nonluminescent gold (I) complexes have usually been considered as useless or worthless by-products. However, in 2016, Xueji Zhang demonstrated an effective route to produce the nonluminescent Au(I)-thiolate complexes for the aurophilic aggregation-induced emission (AIE) for the first time. Cu^2+^ was found to quickly quench the emission of the Au(I)-ligand-Cd^2+^ complexes (Figure 2) and simultaneously cause the Cu^2+^-concentration-dependent redshift of the emission peak wavelength of the Au(I)-ligand-Cd^2+^ complexes, which helped to establish a novel colorimetric sensor for sensitive and selective visual sensing of Cu^2+^ [56]. In 2016, a good sensing of mercury ions in water with high sensitivity and selectivity was reported with the use of gold nanoclusters protected by bovine serum albumin-loaded cellulose nanocrystal-alginate hydrogel beads (Figure 3) [57]. Hg^2+^ was selected for the adsorption experiment and better results were drawn using the linearized form of the Langmuir adsorption isotherm. For the mechanism of fluorescence quenching, it was believed that in the presence of Hg^2+^ there is a high-affinity metallophilic Hg^2+^/Au^2+^ interaction on the Au@BSA NC surface with a visible change in the color because of the adsorption of Hg^2+^ ions by the nanocomposite.

**Figure 4:**
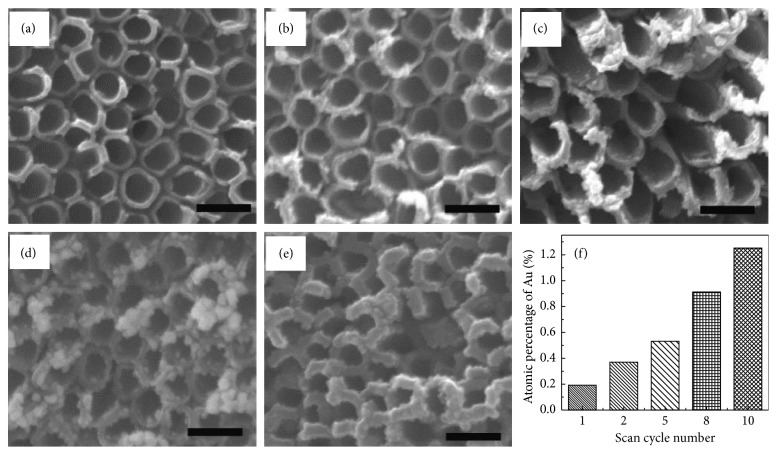
FE-SEM images of the top-view morphology of TiO_2_/Au NTAs (a−e) with different amounts of AuNP loading. The scale bar is 200 nm. (f) Corresponding atomic percentage of Au detected by EDX. (Copyright @2017 American Chemical Society).

In addition to the above research progress, there are many studies on the use of functionalized gold nanoclusters for heavy-metal ion fluorescent probes [58–60]. Except heavy-metal ions, organic anions and organic pollutants may cause a certain degree of environmental impact [61, 62], and some of them have been directly or indirectly proven to have carcinogenic, teratogenic, and mutagenic effects [63]. There is a growing interest in developing high-performance sensors monitoring organophosphate pesticides for their broad usage and harmful effects on mammals. In 2016, M. Reza Hormozi-Nezhad developed a colorimetric sensor array consisting of citrate-capped 13 nm AuNPs which has been proposed for the detection and discrimination of five organophosphate pesticides (OPs). At different pH/ionic strengths, nine AuNPs which they synthesized were employed as simple plasmonic sensing elements in the development of a colorimetric sensor array for the detection and discrimination of five OPs (azinphos-methyl, pirimiphos-methyl, phosalone, chlorpyrifos, and fenamiphos) within the concentration range of 120–400 ng·mL^−1^. They came up with the colorimetric array based on unmodified AuNPs to detect the OPs, and the aggregation behavior of AuNPs against OPs is completely different at different pH and in different ionic strength media. Finally, they successfully applied the sensor array to detect various OPs in real samples [64]. Yang et al. [65] prepared AuNP-modified carbon-doped TiO_2_ nanotube arrays (TiO_2_/Au NTAs) and used TiO_2_/Au NTAs to fabricate a highly sensitive and renewable electrode (Figure 4). With such an electrode, they developed a method for bisphenol A (BPA) detection with higher sensitivity and larger linear range than either the TiO_2_NTAs with UV irradiation or the TiO_2_/Au NTAs without UV irradiation. Without irradiation, a linear range for analytical signals from 0.1 to 28.9 *μ*M was established. The limit of detection (LOD) calculated to be 4.7 × 10^−8^ M (S/N = 3) and a sensitivity of 0.81 *μ*A·*μ*M^−1^·cm^−2^ was achieved. With UV illumination, two linear ranges (0.1 to 3.89 *μ*m and 4.9 to 38.9 *μ*M) were observed and a sensitivity of 2.8 *μ*A·*μ*M^−1^·cm^−2^ and a sensitivity of 0.94 *μ*A·*μ*M^−1^·cm^−2^ were reported correspondingly, with the combination of solid phase extraction and reconstituting the extract containing the target dithiocarbamates into an aqueous dispersion of plain citrate-capped AuNPs. Giokas et. al. described a simple and sensitive nondestructive method for the determination of total concentration of dithiocarbamate fungicides (DTCs) in real samples. In order to improve the detection limits, they scaled down the method to microvolume conditions, avoiding the need to preconcentrate larger sample volumes. With their method (a two-step procedure), the total concentration of dithiocarbamate pesticides was successfully determined in various water samples at the low and ultralow *μ*g· L^−1^ levels, and this method could be used to determine the dithiocarbamate (DTC) fungicide residues in environmental samples [66].

Employing modifiers such as Tiopronin (TPN) [67], 3-mercapto-1,2,4-triazole (TRO) [68], and bovine serum albumin (BSA) [69], our group successfully prepared various gold nanoclusters and applied them to detect melamine, tartrazine, chloramphenicol, and other molecules in samples. This will be of great help in studying the progress of the application and the mechanism of gold nanoclusters in food analysis and environmental analysis. It is the key to perform the rapid analysis of the target pollutants in environmental samples by applying the new principles, new methods, and new technologies to establish the method with high selectivity, rapidness, and sensitivity. Precious metal nanomaterials attract the interests of researchers with their low toxicity and strong fluorescence properties, and the construction of fluorescent probes for such materials has great potential in rapid environmental monitoring applications, and gold nanoclusters are one of them. However, the exploration of fluorescent probes for gold nanoclusters is still under development, and it is necessary to continue studying the large-scale application of fluorescent probes for online detection of real environmental samples. More functional nanoscale fluorescent gold probes are being developed and applied for the detection of real samples, and we can expect that in the near future, the detection of environmental pollutants will be in-site, fast, and convenient.

### 1.2. Application of Gold Nanoclusters in Sensors for Biological Analysis

There are some investigations related to tagging biomolecules and molecular recognition that use the fluorescence probe of gold nanoparticles. According to reports [70], the types of indicators that are selected as standards for the detection is a major challenge for researchers. The number of circulating tumor cells (CTCs) in the blood correlates very significantly with the recurrence of cancer and relapse, and the detection of CTC at low concentrations has a drastic effect on the early and accurate diagnosis of cancer [71]. At the early stage, the concentration of the cancer cells disseminated in blood circulation is very low and it is too difficult to detect them effectively. Therefore, an approach with high sensitivity, rapidness, and specificity is required for the detection of cancer cells at a low frequency in real biological samples. Zhang et al. reported that modified magnetic nanobeads with anti-CD3 could be used as capture probes for efficient and fast magnetic separation of Jurkat T cells from a mixture of cells while gold nanoparticles (AuNPs) conjugated with anti-CD2 could be used as detection probes for ICP-MS measurement. With the optimal conditions, the developed immunomagnetic separation combined with ICP-MS measurement offered the linear range of 300–30 000, a detection limit of 86 Jurkat T cells, and the relative standard deviation for seven replicated detections of Jurkat T cells at 5.2% (3000 Jurkat T cells) [72]. Li et al. [73] designed an ultrasensitive and enzyme-free detection platform for the sensitive detection of the anterior gradient homolog2 (AGR2) based on fluorescence quenching of AuNPs together with the amplification of the hybridization chain reaction (HCR). Two fluorophore-labeled hairpin probes (HP1 and HP2) containing adhesive tails were designed, and the AGR2 aptamer was used as an initiator for the occurrence of HCR between HP1 and HP2 to form a long-cut dsDNA duplex. In the presence of AGR2, the AGR2 aptamer would recognize it specifically and, consequently, could not trigger the occurrence of HCR. As a result, the adhesive tabs of HP1 and HP2 would be adsorbed on the surface of AuNPs to bring the fluorophore to close proximity of the AuNPs and lead to the quenching of the fluorescence signal. AGR2 could be sensitively detected in the range of 5.0 pM–1.0 nM by the established path. It has been reported that the anterior gradient homolog2 (AGR2) is directly associated with a wide range of human cancers, such as cancer of the esophagus, pancreas, breast, prostate, and lung [74, 75].

**Figure 5:**
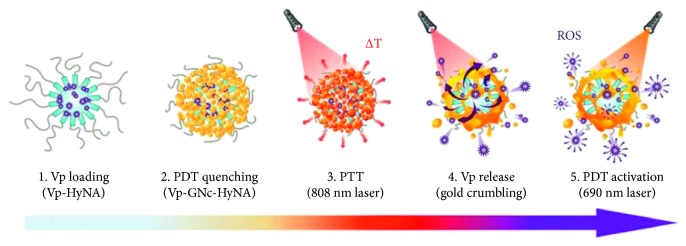
Schematic illustration of the Vp release mechanism from Vp-GNc-HyNA by sequential PTT/PDT laser treatment. (Copyright @2017 American Chemical Society).

The aforementioned result is based only on the detection of certain small cancer-related molecules in the body and has not considered the possibility of the use of gold nanoclusters to treat or control the tumor. Han et al. [76] demonstrated that a biostable, photo- and enzyme-activated hybrid nanomaterial based on a gold nanocluster (HyNA) could be used as a potential photothermal therapy (PTT) agent and as a photothermally activatable smart photodynamic therapy (PDT) nanoformulation using Vp-GNc-HyNA (Figure 5).

This whole model of gold nanoclusters was selectively installed on the outer shell of the hyaluronan nanoassembly to form a gold shell. Because of the dual protective effects from both the hyaluronan self-assembly and the inorganic gold shell, outstanding stability of verteporfin-encapsulated GNc-HyNA (Vp-GNc-HyNA) in the bloodstream was observed. Surprisingly, the fluorescence and photodynamic properties of Vp-GNc-HyNA were significantly quenched owing to the gold nanoclusters covering the surface of the nanoassemblies; however, photothermal activation by 808 nm laser irradiation prompted a substantial rise in temperature, which induced the PDT effect of Vp-GNc-HyNA. They claimed that Vp-GNc-HyNA might present a great potential to treat tumors both in vitro and in vivo. Their results showed that the tumors were entirely ablated with a 100% survival rate and complete skin regeneration during the 50 days following treatment with Vp-GNc-HyNA in an orthotopic breast tumor model [76].

In addition, detection of microRNAs, DNA of pathogenic bacteria, and other small molecule has also made great progress based on the function of gold nanocluster materials on the life of pyrophosphatase.

The research and testing of various life activities within the organism is an important way to explore the relationship between internal life activities and biological health. DNA plays an important role in gene therapy, clinical diagnosis, and mutation analysis. Due to a variety of factors, there may be the phenomenon of base misdiagnosis in the process of DNA transcription and translation [77]. Zhou et al. introduced binary DNA probes into an AuNP-based colorimetric system and discovered visual discrimination of single-nucleotide variation in the target DNA. Moreover, they found that the ability of discrimination of single-base mismatch was greatly improved by the utilization of the binary DNA probes instead of the intact long-probe DNA, which holds great potential for increasing the accuracy of disease diagnosis in clinical applications [78]. Like DNA chains, the active intracellular substances will also affect the organism; we can study and explore the relationships between the cellular life and these substances from the mechanism and function by fluorescent labeling. [79, 80]. Due to the high toxicity of traditional fluorescent labeling dyes, poor biocompatibility, and instability in fluorescence, it is imperative to develop a new fluorescent material. The unique optical properties of gold nanoclusters make it possible for them to become a new fluorescent probe, especially when gold nanoclusters are functionally modified and have different shapes and functions. Current diagnostic approaches, such as enzyme-linked immunosorbent assay (ELISA) [81], enable the capture and detection of proteins. Scott et al. synthesized novel biotin-polyethylene glycol (biotin-PEG) gold nanoparticle probes and used them for the detection of protein (prostate-specific antigen, PSA) and nucleic acid targets (microRNAs) from a single sample, and it might be useful for the detection and management of early-stage diseases [82].

**Figure 6:**
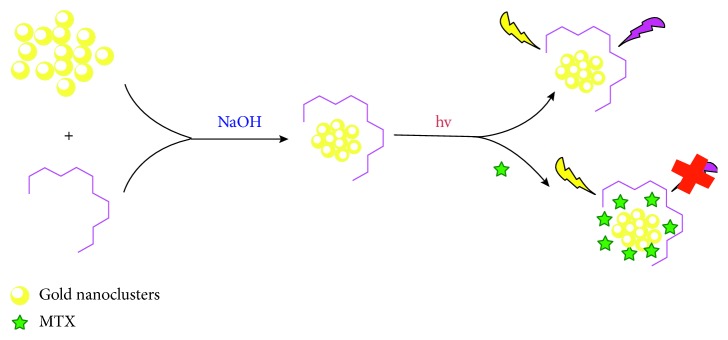
Schematic illustration of the fluorescent BSA-AuNCs for MTX detection.

Similarly, methotrexate (MTX) is an antimetabolite used in the treatment of some cancers of the breast, head, neck, and skin. MTX has been measured using electrochemical methods [83], high-performance liquid chromatography (HPLC) [84], liquid chromatography (LC) [85], liquid chromatography/mass spectrometry (LC/MS) [86], liquid chromatography/mass spectrometry/mass spectrometry (LC/MS/MS) [87], surface-enhanced Raman spectroscopy (SERS) [88] and so on. Chen et al. developed a method to detect methotrexate using gold nanoclusters protected by bovine serum albumin as the fluorescence probes for the first time [89], in which the fluorescence intensity of the gold nanoclusters was decreased after the addition of methotrexate (Figure 6). The method provided the range of 0.0016 mg·mL^−1^ to 24 mg·mL^−1^ and the detection limit for methotrexate at 0.9 ng·mL^−1^ at a signal-to-noise ratio of 3. General fluorescent imaging probe reagents are organic dyes, green fluorescent protein, and inorganic quantum dots, but they possess toxicity, lower molar absorption coefficient, and lower antilight bleach toxicity, and they will cause serious pollution of the biological environment when used for cell imaging. Chen et al. established the tumor-targeting imaging using dual fluorescence nanoconjugates based on gold nanoclusters, and the nanoconjugates exhibited dual fluorescence in the visible range and NIR range simultaneously because of the composition of the cyclic (Arg–Gly–Asp–DTyr–Lys) (cRGD) peptide and a near-infrared (NIR) fluorescence dye (MPA). They demonstrated the outstanding tumor-targeting capability of Au–cRGD–MPA for tumor cells through cell affinity investigations and elucidated the biodistribution characteristics and tumor-targeting ability of the probes in tumor-bearing mice and normal nude mice, respectively. Finally, for the effect of small size (less than 5 nm), most nanoconjugates are metabolized from living subjects via the kidney-bladder route and are therefore not harmful to organisms [90]. Yin et al. developed a gold nanoparticle probe-based assay (GNPA) for ultrasensitive detection of the hepatitis C virus (HCV) core antigen [91]. In addition to the development of several DNA enzyme-based sensors for application in living cells, it has also made a certain degree of research progress in intracellular aptamer-related research. Huang et al. developed the aptazyme sensor for amplified molecular probing in living cells by using gold nanoparticles (AuNPs) modified with substrate strands hybridized to aptazyme strands [92]. In the study, Lee et al. successfully produced graphene sheets decorated with AuNPs (AuGrp) using phytochemicals as reducing agents, and Au ions were intercalated into the layered graphene flakes and were then reduced into NPs, exfoliating the graphene sheets. Using antibody (Ab)-modified AuGrp sheets and quantum dots, a plasmonic-induced photoluminescence immunoassay of the tuberculosis (TB) antigen (aG) CFP-10 was demonstrated for a potential application of these materials [93]. Recently, we also used 2-mercapto-4-methyl-5-thiazoleacetic acid (MMTA) as a protective agent to successfully synthesize monomolecular-protected gold nanoclusters (MMTA-AuNCs). The particle size of the gold nanoclusters prepared by the synthesis method is relatively uniform, and the average particle size is 4 nm with better water solubility and higher stability. The interaction between MMTA-AuNCs and bovine serum albumin was studied by fluorescence spectroscopy. The results showed that the quenching mechanism of gold nanoclusters on bovine serum albumin was mainly static quenching.

## 2. Conclusions

Functional gold nanoclusters will play an increasingly important role in the application of biological imaging, fluorescent labeling, and other aspects. Gold nanoclusters have been widely used in the fields of fluorescent probes and biomarker imaging in recent years, due to their specific optical properties. The use of fluorescence probes has demonstrated the potential on the recognition and fluorescence labeling of DNA molecules, proteins, and small molecules based on the functionalized gold nanoclusters. Currently, the research of fluorescent probes based on gold nanoclusters is based on the fact that the structure of the nanomaterials is not clear; however, the effect of the structure of the nanometric material on its properties is very important. In future studies, we hope that it will be possible to precisely control the synthesis of the internal structure of gold nanoclusters for functional purposes.
